# Critical analysis of moderate and severe retractions in the pars tensa and pars flaccida of the tympanic membrane^⋆^^⋆⋆^

**DOI:** 10.1016/j.bjorl.2021.10.005

**Published:** 2021-11-15

**Authors:** Inesângela Canali, Letícia Petersen Schmidt Rosito, Vittoria Dreher Longo, Sady Selaimen da Costa

**Affiliations:** a Universidade Federal do Rio Grande do Sul, Porto Alegre, RS, Brazil; b Universidade Federal do Rio Grande do Sul (UFRGS), Hospital de Clínicas de Porto Alegre (HCPA), Departamento de Otorrinolaringologia – Cirurgia de Cabeça e Pescoço, Porto Alegre, RS, Brazil; c Universidade de Caxias do Sul - Rio Grande do Sul, Porto Alegre, RS, Brazil

**Keywords:** Tympanic membrane retraction, Pars flaccida, Pars tensa, Air bone gap

## Abstract

**Objectives:**

Analyze the prevalence of retractions in different areas of the Tympanic Membrane (TM), the correlations between the involvement of the Pars Tensa (PT) and Pars Flaccida (PF), and the air-bone gaps.

**Methods:**

A cross-sectional study. Patients with moderate and/or severe TM retraction of 2200 consecutive patients with chronic otitis media between August 2000 and January 2019 were included. Ears with previous surgery were excluded. Ears were classified as isolated PF and PT retractions and association of both. The degrees of severity and presence of effusion were evaluated. The data were analyzed using the SPSS Statistics software program.

**Results:**

661 ears were included. The prevalence of isolated atical retractions was 24.9%, of isolated posterior quadrants was 10.6%, and of association of quadrants was 64%. There was no correlation between the retractions in the different areas of the TM (posterior and attic quadrants: *r* = 0.13; *p* = 0.041; anterior and posterior quadrants: *r* = 0.23; *p* = 0.013, anterior and attic quadrants: *r* = 0.06; *p* = 0.043). Effusion was present in 30.7% of the ears. ABG median was lower in ears with PF retraction (6.25 dB HL) than PT retraction, isolated (15 dB HL) or not (13.75 dB HL; *p* < 0.05); 72% of the ears had an ABG ≤ 20 dB HL. For severity of the retraction of PF, the ABG was similar across groups. For the PT, there was a global difference in the medians of ABG in terms of the degree of severity, with a moderate correlation.

**Conclusion:**

The prevalence of moderate and severe retractions was 24.5%; 64% of the ears had an association of affected regions. There was no correlation between the retraction in the different areas of the TM. We found a significant correlation between the severity of retraction and the worsening of ABG threshold, only for PT.

**Evidence level:**

4.

## Highlights

• The literature is scarce in demonstrating the correlation between the degree of severity of retractions and the degree of hearing loss.
• To analyse the differences in the location, the severity, and the air-bone gap of moderate and severe tympanic membrane retractions.
• After a global analysis of the behavior these retractions, we observed the need for classifications that evaluate them globally.
• Identification of these gaps in literature is relevant so that the focus may be shifted on these topics and proper studies may be implemented.

## Introduction

Tympanic Membrane (TM) retraction is defined as the partial or total inward displacement of the Pars Flaccida (PF) or Pars Tensa (PT) of the membrane towards the promontory.^1^

There are many classifications in literature for the degrees of severity of the retractions. Sadé and Berco (1976) were the first to propose the best-known classification for the degree of severity of retraction.^2^ Since then, modified classifications to define severity based on variations have been proposed by several authors. The following parameters were considered: topography, presence of ossicular chain erosion, self-cleaning property, and TM atelectasis.^3^ Despite being didactic, they only describe a momentary state of TM segments and the middle ear, and they do not define prognosis or management.

It is believed that the formation of TM retractions is multifactorial. First, PF would be a region inherently susceptible to natural fragility due to the histological characteristics of its fibrous middle layer with loss of connective tissue and collagen bundles in this area.^1^ Second, it is believed that retractions of PT would be the result of chronic inflammatory processes resulting from a continuum, triggered by a sustained tubal dysfunction.^3^, ^4^ Third, other factors such as the deregulation of the ventilation pathways of the middle ear-mastoid complex are involved. Blocking the two main airways in the Prussak space, the anterior and posterior isthmus, can compartmentalize the areas of the middle ear cleft, creating selective epitympanic ventilation, predisposing to the formation of retractions.^5^, ^6^, ^7^ This can occur focally in the epitympanum and postero-superior quadrant, or globally, in all TM.

Epidemiological studies on the prevalence of retractions are scarce in literature. The lack of a standardization in its classification makes it difficult to compare the various studies and, consequently, to determine the real prevalence taking into account the degree of severity and the region of the TM affected. In addition, the retractions were classified in literature systematically, according to the TM's regions affecting PF and PT. Clinically, however, we observed that retractions usually affected more than one region at the same time in most cases.

The objectives of this study, therefore, were: 1) To analyze the prevalence of retractions and compare their frequencies in different areas of the TM; 2) To analyze the degree of severity in the different regions; 3) To analyze the prevalence of the concomitance of the regions involved; and 4) To evaluate the audiometric pattern in these ears.

## Methods

This cross-sectional study enrolled a sample of 2200 patients with Chronic Otitis Media (COM) who were followed up at a tertiary hospital between August 2000 and January 2019. The inclusion criteria were the presence of moderate or severe retraction of the TM in at least one ear. The exclusion criteria were the presence of mild TM retraction, history of previous otological surgery (except myringotomy for ventilation tube insertion), inability to videotoscopy for adequate documentation, and refusal to participate in the study. Patients with residual TM perforation were excluded.

**Figure 1:**
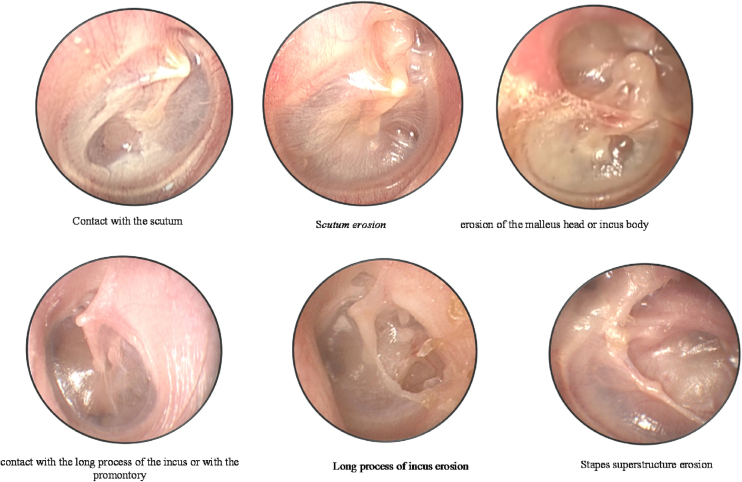
Classification according to the modified Sadé and Berco classification by our group.^6^ (A) Pars flaccida-contact with the scutum; erosion of the scutum (attical lateral wall); erosion of the malleus head or incus body. (B) Pars tensa-posterior quadrant: contact with the long process of the incus or with the promontory; erosion of the long process of the incus; erosion of the stapes superstructure.

During the first visit, the patient's clinical history was collected, and both ears were examined with microscopic analysis and videotoscopy with a fiber-optic otoscope (0° and 4-mm otoscope; Karl Storz GmbH, Tuttlingen, Germany). The otoscopic findings were recorded for analysis and systematically described according to the classification protocol by the same otologist, to avoid bias. The retractions were classified according to the modified Sadé and Berco classification by our group.^6^ Moderate retractions were considered when there was contact of the TM on the ossicular chain or in the promontory, and severe retractions when there was erosion of the ossicular chain or bone limits. TM was divided into PF (atticus) and PT (posterior and anterior quadrant). Each region was analyzed, and the following degrees of severity were noted (Fig. 1):

PF: 1, normal; 2, contact with the scutum (modest retraction in the attical lateral wall without contact with the malleus neck and/or ossicle heads or erosion); 3, erosion of the scutum (attical lateral wall); 4, erosion of the malleus head or incus body.

PT – Posterior Quadrant (PQ): 1, normal; 2, contact with the long process of the incus or with the promontory; 3, erosion of the long process of the incus; 4, erosion of the stapes superstructure.

Grade 2 was considered moderate retraction while Grade 3 and 4 severe, for both PF and PT (PQ).

PT – Anterior Quadrant (AQ): 1, normal; 2, contact with the promontory.

Grade 2 was considered moderate retraction.

The presence of effusion in the middle ear during videotoscopy was evaluated in all the ears studied. Since there is no standardization of the TM mobility assessment methodology, we chose not to analyze this variable in the present study.

All patients underwent pure-tone and speech audiometry to determine the Air-Bone Gap (ABG), Air Conduction (AC) and Bone Conduction (BC) thresholds using the AD 27 audiometer (Interacoustics AS, Assens, Denmark) and THD-39 supra-aural earphones (Telephonics Corporation, Farmingdale, NY, USA). The ABG was calculated as the difference between the AC and BC. We described AC, BC, and ABG using the 4-frequency Pure-Tone Average (PTA), calculated by averaging the thresholds at 500, 1000, 2000, and 4000 Hz. For audiometric analysis, we exclude ears with effusion, since this could be a confounding factor in the size of the conductive hearing loss.

Patients under 18 were considered children, according to the criteria of the World Health Organization.

This study was approved by the ethics committee (protocol 160258) and conformed to the tenets of the Helsinki Declaration. A consent form was signed for the anonymous use of patient data. Parents or guardians of the children signed informed consent forms for enrollment. The treatment was not influenced by whether the patients participated in the study or not.

Data were stored in a database and the Statistical Package for Social Science (SPSS, version 22) was used for statistical analyses. Quantitative variables were described as means and Standard Deviations (SD). Categorical data were described as counts and percentages. Gamma and Spearman correlation coefficients were used to describe the correlation between the alterations. Non-normal quantitative data were described by the median, minimum, and maximum values. *t*-test and analyses of variance were used to compare normally distributed data. Mann-Whitney and Kruskal-Wallis tests were used for asymmetric data. The level of significance was set at *p* < 0.05.

## Results

Among the 2200 patients with COM studied, we included 540 patients (24.5%) with moderate or severe TM retraction in at least one ear. The mean age of the related patients was 32 years, SD ± 20 years (4–81 years), and 54% were female. Hypoacusis was reported in 43.2% of the patients, otorrhea in 36.4%, and otalgia in 15%.

Since 121 patients (18.3%) presented this alteration bilaterally, we studied a total of 661 ears. Two hundred and eighty (42.4%) were from pediatric patients and 381 (57.6%) were from patients aged 18 years or over (*p* > 0.5). Regarding laterality, 54% of the ears were right. A previous history of myringotomy for the ventilation tube was observed in 13.8% of the cases. Effusion was present in 30.7% of the ears included in the study.

**Figure 2:**
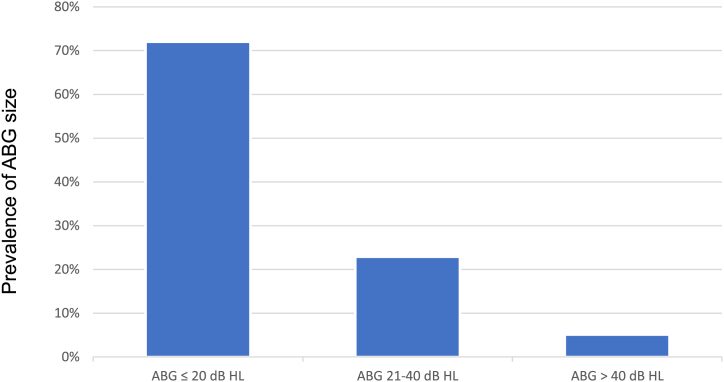
Prevalence of the Air Bone Gap (ABG) size.

Audiometry was performed in 394 ears (87.3%) out of 451 without effusion. For the AC, BC, and ABG thresholds, the median (minimum – maximum) of the PTA was 25 dB HL (0–120 dB HL), 10 dB HL (0–75 dB HL). and 12.5 dB HL (0–55 dB HL), respectively. The prevalence of ABG size (PTA) in the population is shown in Fig. 2. There was no significant difference in ABG threshold in patients with unilateral vs. bilateral retractions (ABG thresholds median 10 bB vs. 13.3 dB, respectively; *p* = 0.64).

### Analysis of each region of the tympanic membrane retractions

The attical region was affected in 563 ears (85.1%), the posterior quadrants in 491 ears (74.2%), and the anterior quadrants in 117 ears (17.7%).

When analyzing the exclusive alterations of each area, we observed that 164 ears (24.9%) presented isolated attical retraction, 70 ears (10.6%) presented isolated posterior quadrant retraction, and two ears (0.3%) presented isolated anterior quadrant retraction. Sixty-four percent (425 ears) showed changes in more than one quadrant at the same time.

#### Isolated retractions of the pars flaccida (atticus)

**Figure 3:**
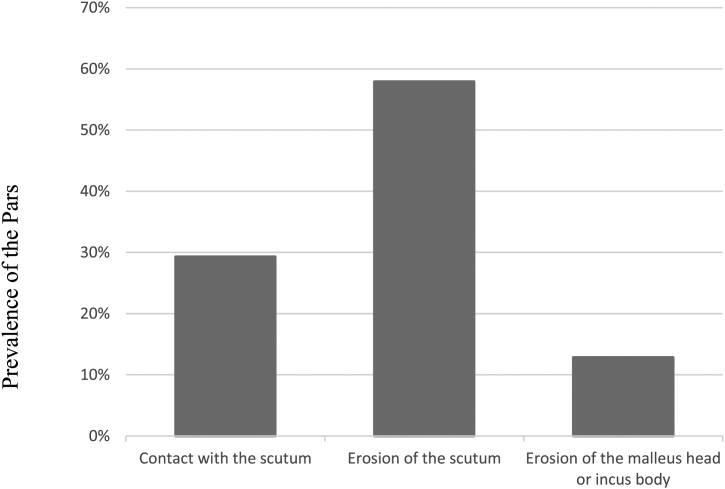
Prevalence of isolated retractions in the pars flaccida (atticus).

When analyzing the 164 ears with isolated attical retractions, we observed the following prevalence, as shown in Fig. 3.

Audiometry was performed in 103 (88%), of the 117 ears with exclusive PF alterations, without effusion. Among these, the PTA median (minimum – maximum) was 22.5 dB HL (0–85 dB HL) for AC, 13.7 dB HL (0–55 dB HL) for BC, and 6.2 dB HL (0–60 dB HL) for ABG.

**Figure 4:**
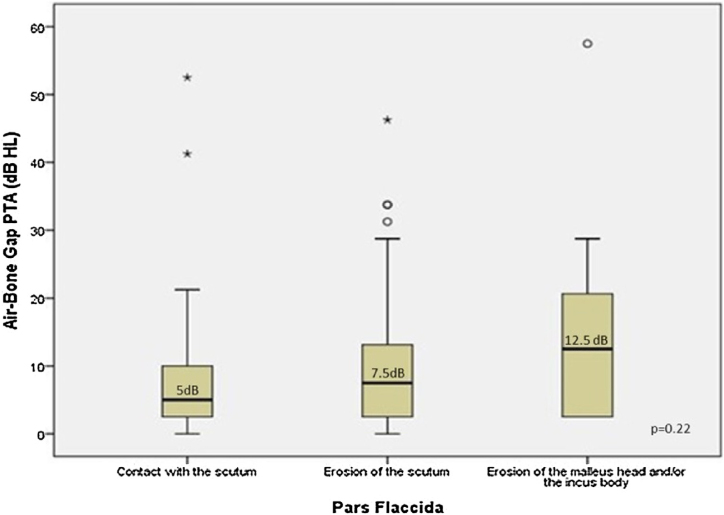
Comparison of the Air-Bone Gaps (ABGs) at the PTA (dB HL) related to the severity of the isolated retractions in PF. The boxes' central horizontal lines and superior and inferior limits indicate median values and interquartile ranges (75th and 25th percentiles), respectively.

Fig. 4 presents the patterns of ABG PTA medians compared to the severity of alterations in PF. The three groups of severity (contact with the scutum; erosion of the scutum; erosion of the malleus head or of the incus body) were similar in the dispersion of the bar graphs for both ABG (Kruskal-Wallis test: ABG PTA median 5 dB HL; 7.5 dB HL; 12.5 dB HL respectively; *p* = 0.22). The Spearman correlation was weak (*r* = 0.17, *p* = 0.08) (Fig. 4).

#### Isolated retractions of the pars tensa (posterior quadrant)

**Figure 5:**
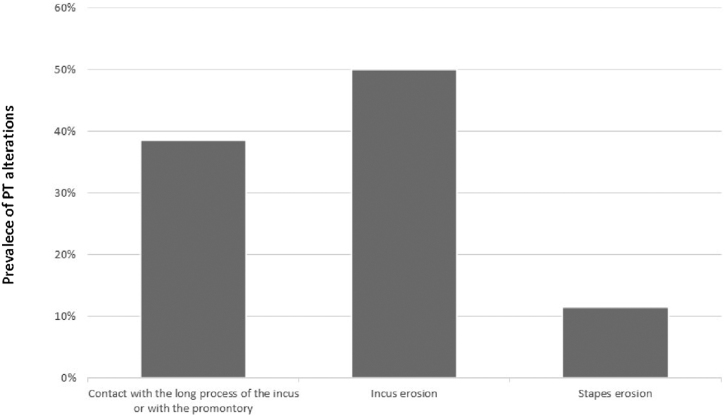
Prevalence of isolated retractions in the posterior quadrants.

When analyzing the 70 ears with isolated retractions of the posterior quadrants, we observed the following prevalence, as shown in Fig. 5.

Of the 42 ears with isolated PT retractions without effusion, audiometry was performed in 35 ears (83.3%). Among these, the AC PTA median (minimum – maximum) was 18.7 dB HL (5–100 dB HL), BC 7.5 dB HL (0–70 dB HL), and ABG 15 dB HL (0–45 dB HL).

**Figure 6:**
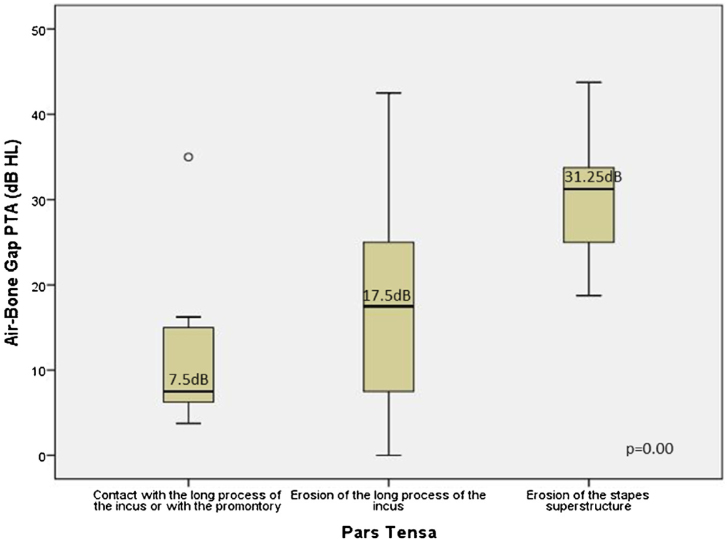
Comparison of the Air-Bone Gaps (ABGs) at the PTA (dB HL) related to the severity of the isolated retractions in PT (posterior quadrant). The boxes' central horizontal lines and superior and inferior limits indicate median values and interquartile ranges (75th and 25th percentiles), respectively.

Fig. 6 an illustrates a global difference between the ABG PTA medians and the severity of retraction (contact with the long process of the incus or with the promontory, erosion of the long process of incus, and erosion of the stapes superstructure) (Kruskal-Wallis test: median 7.5 dB HL; 17.5 dB HL; 31.25 dB HL, respectively; *p* = 0.01). This difference occurred between ears having contact with the TM in the long process of the incus and/or promontory and ears with an erosion of the long process with the incus (*p* = 0.04) and with an erosion of the superstructure of the stapes (*p* = 0.00), and also between incus erosion and stapes erosion (*p* = 0.00). We also observed a direct and moderate correlation of severity and worsening of the ABG PTA (Spearman coefficient *r* = 0.5, *p* = 0.00).

#### Anterior quadrants

Only 17.5% (117 ears) had alterations (contact with the promontory) in the anterior quadrants at the same time as alterations in other regions, and only two ears had exclusive anterior alterations.

### Concomitances of alterations in retractions in the tympanic membrane regions

Table 1 shows the frequency of alterations in each region when isolated and when associated with alterations in other areas. We observed that the proportion of isolated PF retractions was significantly higher than that of isolated PQ (*p* < 0.001). On the other hand, AQ retractions rarely occurred isolated.

**Table 1:** Frequency of alterations in each region when isolated and when associated with alterations in other quadrants.

Area of retraction	Concomitance of retractions	Frequency
PF retractions (n = 563)	Isolated	29.1%
	Association with QP	70.1%
	Association with QA	15.6%
PT – QP retractions (n = 491)	Isolated	14.2%
	PF association	80.4%
	Association with QA	22.4%
PT – QA retractions (n = 117)	Isolated	1.7%
	PF association	75.2%
	Association with QP	94%

Correlation between the location of the retraction in the attic and the QP or between the attic and the QA was not observed (Spearman coefficient *r* = 0.13; *p* = 0.041 and *r* = 0.06; *p* = 0.043, respectively). However, was observed a weak correlation between PQ and AQ impairments (*r* = 0.23; *p* = 0.013).

## Discussion

We observed a significant prevalence of moderate and severe TM retractions, corresponding to 24.5% of all patients with COM. However, in literature, it varies between different studies. Sudhoff et al. (2000) studied children who underwent adenoidectomy and myringotomy for ventilation tubes, observed a prevalence of severe posterosuperior retractions of 3.2%–5.1%.^8^ Maw et al. (2011), on the other hand, observed a frequency of 8%–10% in children, although mild retractions were included.^9^ Other authors also observed that the prevalence of moderate and severe retractions such as atrophy of the PT in children, varying between 0.7% and 10%.^10^ However, studies on TM retractions in adults are scarce.

Although there are many studies on retractions, they are not subject to comparisons, since the inclusion criteria are not the same and the classifications are different, and, therefore, are not in agreement with one other.

The introduction of endoscopes for ear examination in the recent decades, has allowed a clearer view of the attic region and bone erosions of the postero-superior quadrant. The presence or not of adhesions of TM to the ossicular chain or promontory, however, remains a challenge. The lack of standardization in the technique used for its evaluation, the intra- and inter-individual differences in the performance of the Valsalva maneuver, the difficulty of the pediatric population (which corresponded to 42% of our sample), and the lack of correlation between the data observed clinically and those found during surgery after the use of protoxide make its evaluation extremely difficult and of little use. It appears that the presence of erosions of the ossicular chain or the accumulation of epithelial debris in the retraction (cholesteatoma) are the main indicators of severity.

The retractions were classified in the literature systematically, according to the TM's regions affected into PF and PT. In the present study, we analyzed the behavior of retractions in all areas of TM (attic, QP, and QA) and observed that the majority of retractions have an association of affected regions. This finding suggests that, although didactic, the current classifications do not describe the retractions globally and, therefore, are not able to present us with an accurate clinical diagnosis. In addition, the last stages of some classifications represent the presence of cholesteatoma. We know that the distinction of a severe retraction, starting the formation of epithelial debris with a cholesteatoma, is often a challenge.

The region with the highest prevalence of impairment was the attic followed by the posterior quadrants. A minority had compromised the anterior quadrants. We also observed that 24.8% of the ears had exclusively PF changes, 10.6% exclusively of posterior quadrants, and only two ears of anterior quadrants alone. The prevalence of PF isolated retractions observed in our study can be explained by the pathophysiology of middle ear ventilation pathways. The blocking of the tympanic diaphragm, due to the retraction itself or due to an inflammatory process, with thickening of the mucosa, can cause poor ventilation in the middle ear, creating the selective epitympanic dysventilation syndrome, or even global, as the negative pressure is present and other areas of TM are affected.^7^, ^11^ The PF retraction would therefore be a consequence of the blockage of the epitympanic diaphragm by a preexisting retraction in the PQ. As the ventilation of the mesotympanum would be more easily restored, either by resolving the inflammatory processes of the middle ear or restoring the functioning of the auditory tube, the blockages of the anterior and posterior isthmus, which compartmentalize the epitympanic space, could be more difficult to resolve naturally. We observed in the transoperatory that such isthmus is routinely obliterated in the attic cholesteatomas, either by fibrosis or by granulation tissue. Thus, retractions would occur concurrently in both regions most of the time and because ventilation in the middle ear has a greater tendency to spontaneously reestablish, changes in PQ would resolve more easily, explaining the higher prevalence of retractions in the PF when seen isolated.

The most frequently observed alteration in the PF was erosion of the scutum, whereas in PQ, it was erosion of the incus, suggesting that PQ retractions would be more frequently associated with conductive hearing loss. A previous study by our group has shown that incus erosion is the alteration associated with larger air-bone gaps in posterior mesotympanic cholesteatomas when compared with posterior epitympanic.^12^

We found a significant correlation between the severity of retraction and the worsening of ABG threshold, only for PT. This finding indicates that ABG measure is not influenced by even more severe PF involvement. However, the severity of ossicular erosion in the posterior quadrants was predictive of an increase in the ABG. The significant repercussions of the degree of hearing loss in these cases depend on the decision whether the patient will benefit from early intervention or not. As described in the literature, we know that stapes superstructure erosion is one of the main predictors of poor surgical results; therefore, the probability of a favorable post-surgical outcome is uncertain in patients with small ABG.

A review of studies on surgical indication and hearing results of ossiculoplasty revealed that the minimum preoperative ABG required ranges from 24 to 27 dB HL. The American Academy of Otolaryngology Committee on Hearing defines treatment success as conductive hearing loss equal to or lower than 20 dB HL.^13^ The rates of treatment success ranged from 47% to 86.9% with Total Ossicular Reconstruction Prosthesis (TORP) and from 50% to 84.6% with Partial Ossicular Reconstruction Prosthesis (PORP).^14^, ^15^, ^16^, ^17^ All studies agree that the presence of stapes determines a better prognosis in the results of ossiculoplasty,^16^ since it does not provide additional audiometric gain, but promotes better stabilization of the prosthesis.^18^ Thus, reconstruction of the ossicular chain would not be justified in retraction cases with an ABG lower than 25 dB HL, especially in patients with stapes erosion. However, factors such as the presence or progression to cholesteatoma, and the frequency of ear infections must be considered when monitoring therapies.

In literature, there are no studies that show a correlation of the concomitance of retractions in the various segments of TM. Apparently, the quadrants show individual behaviors; thus, we can find significant alterations of the posterior quadrants, with normal PF, and vice versa. The only association that showed a mild correlation with each other was that when the anterior quadrant was compromised, the posterior quadrant was also affected.

The limitation of this study is to know which ones of these retractions will be stable for years and which will evaluated for cholesteatoma and conductive hearing loss. For that well-conducted cohort studies are needed to identify the risk factors for this behavior. In addition to that, the staging systems should take into account the auditory status, thus being more functional and being able to direct conduct.

After a global analysis of the behavior of moderate and severe retractions in the TM, we observed the need for classifications that do not sector the retractions in the TM but evaluate them globally, associating the audiometric pattern.

## Conclusion

Moderate or severe retractions of TM corresponded to 24.5% of all the cases of COM in the study. 64% of the ears presented retraction in more than one region. We found no significant correlations between changes in the various TM quadrants. 72% of the ears presented an ABG less than 20 dB HL. The ABG PTA median was higher when the PQ were involved. There was a significant correlation between the severity of the retraction and the worsening of the ABG only in the isolated PT retractions.

## Conflicts of interest

The authors disclosure no conflicts of interest or funding received for this work from any of the following organizations: National Institutes of Health (NIH), Wellcome Trust, Howard Hughes Medical Institute (HHMI), and other(s).
